# Distress Due to Urinary Problems and Psychosocial Correlates among Retired Men in Hong Kong

**DOI:** 10.3390/ijerph17072533

**Published:** 2020-04-07

**Authors:** Marcus Yu Lung Chiu, Ho Ting Wong, Xue Yang

**Affiliations:** 1 Department of Social and Behavioural Sciences, City University of Hong Kong, Tat Chee Ave, Kowloon Tong, Kowloon, Hong Kong, China; 2 Institute of Health Care Management, Department of Business Management, National Sun Yat-sen University, Kaohsiung 80424, Taiwan; frankwong@connect.hku.hk; 3 JC School of Public Health and Primary Care, Faculty of Medicine, The Chinese University of Hong Kong, Prince of Wales Hospital, Shatin, Hong Kong, China; sherryxueyang@cuhk.edu.hk; 4 The Chinese University of Hong Kong Shenzhen Research Institute, Shenzhen 518172, China

**Keywords:** ageing, urinary incontinence, nocturia, masculinity

## Abstract

Urinary problems are common among aging men, but there is a paucity of research efforts to understand the psychosocial aspects of the illness. This study aims to understand how common and distressing urinary problems are for newly retired men in Hong Kong and to test the associations between mental health, self-stigma of seeking help, fatigue, self-efficacy, self-esteem, and distress due to urinary problems. To assess this, 139 out of 200 members of a retired men’s social club (mean age 63.5) were successfully interviewed. Two-fifths of the participants felt distressed due to their urinary problems and one-third of the participants had been troubled by urinary incontinence or nocturia in the past six months. Yet the majority of the participants (55%) did not seek help from any medical profession. The group who were distressed by urinary problems showed significantly poorer mental health, reported more fatigue symptoms, were less satisfied with their sexual relationships and overall self-esteem, and were less able to stop unpleasant thoughts or to get social support than the non-distressed group. Cultural perceptions of masculinity and decreased sexual vigor might have affected participants’ willingness to seek help at an early stage. Targeted health education, mutual support groups, and sensitively designed services at the community level are suggested to address these physical and mental health issues.

## 1. Introduction

Urinary problems, commonly caused by “Benign Prostatic Hyperplasia” [1,2,3], comprise of a mixture of symptoms from three categories: storage (symptoms such as nocturia or urinary incontinence), voiding (symptoms include reduced urinary stream or terminal dribble), and post-micturition (symptoms such as the sensation of incomplete emptying) [1,4].

Urinary problems are considered common among older men in Western and Asian countries [1,5] and men are more likely than women to suffer from them [4,6]. Furthermore, the prevalence and the severity of urinary problems tend to increase with the age of men [1,4]. A study in Sweden, the United Kingdom, and the United States reported that approximately half of the men in a community sample aged ≥40 experienced urinary incontinence in the past month (sample size n = 14,140) [5]. Hong Kong studies [7,8] also revealed findings comparable to Western and other Asian countries. In Wong, Woo, Hong, Leung, Kwok, and Leung’s [7] study, 1739 older Chinese men (above age 65) were openly recruited from the community and assessed on “Lower Urinary Tract Symptoms” (LUTS) using the Chinese version of the International Prostate Symptom Score (IPSS) scale. Results showed that out of a sample of 1739 older men, 56.4% had a mild level of LUTS, 32.2% a moderate level, and 6% a severe level. A more recent study [8] also analyzed data from a community sample of 319 Chinese men aged above 40, who were recruited through local media advertisements, and revealed that 30.7% had zero to mild LUTS, 43.9% moderate LUTS, and 25.4% severe LUTS.

Findings from both Hong Kong studies, possibly the only existing ones, warn of a high prevalence of a moderate to severe level of urinary problems among older Hong Kong Chinese men [7,8]. There may be a higher prevalence if there was a self-selection bias in recruiting healthier or more interested research participants in both studies. In addition, the studies also showed that with increases in age, the proportion of men with moderate to severe LUTS increases significantly [7,8]. Herein, the above implications draw attention to the need for studies to look into older men with urinary problems to increase knowledge that will inform both professional practice and service providers.

Although urinary problems, in themselves, are not life threatening [9], the impact on physical health, psychosocial functioning, daily life activities, and quality of life is substantial [10,11]. The seriousness of consequences may range from embarrassment over frequent toilet going [12], actual leakage [13], declined performance in daily life activities to general health issues such as sleep loss and daytime fatigue [10,14,15,16], and may cause psychological and emotional distress [17]. It may even discourage older men with urinary problems from leaving home and exclude them socially [12,18]. For men, urinary problems are stigmatizing [12] and can diminish their masculine identity [19], resulting in internalization of negative self-worth and low self-esteem [17,20]. The impact on spouses, such as affecting their sleep and reducing sexual and marital satisfaction, has also been documented [21,22]. However, the mention of one’s urinary problem in daily conversation is culturally tabooed and therefore, invisible to social relationships.

Urinary problems have been found to be significantly associated with increased depression or anxiety in previous studies [5,11,23,24,25]. In a sample of Chinese men aged 62–92 in Hong Kong, using the Geriatric Depression Scale [19], the study found that nearly two-thirds (64.7%) of men who had “moderate to severe” LUTS were also depressed. Studies on the relationship between urinary problems and mental health issues in Chinese men in Hong Kong are few in number [26,27] and there is a need to better understand the impact of urinary problems on general psychological functioning in Hong Kong men. This study is one of the only few studies that has focused on general mental health status and distress in a sample of Chinese men. An understanding of the psychological functioning of these men would support helping professionals with urinary problems in their work.

Urinary problems as a stressor may reduce ones’ coping self-efficacy. Individuals with urinary problems may limit their social activities outside of the home and develop a restricted and isolated lifestyle [17,28,29]. Embarrassment and shame related to incontinence can lead to a poor self-concept and a lower sense of self-control [17,30,31]. Thus, individuals distressed by urinary problems may have poorer coping resources and coping capacity and thus, a lower coping self-efficacy.

Urinary problems may increase fatigue symptoms. “Fatigue” has been defined as the “sense of persistent tiredness or exhaustion that is often distressing to the individual”. It is multidimensional and includes physical, mental, and emotional tiredness [32] (p.191). Fatigue is a common consequence of chronic illnesses such as cancer, asthma, and stroke [32,33] and has a negative impact on QoL and psychosocial and daily functioning [34]. Some studies have suggested that urinary problems as a chronic disease condition may cause fatigue because some symptoms (e.g., urinary leakage, frequent voiding at night) increase difficulties in daily life activities and sleep loss [10,14,15,16].

To understand the impact of urinary problems, the wider social and cultural contexts that older men live in should be taken into account [35]. For urinary problems, behaviors or characteristics arising from them such as urinary leakage, urinary stains and odor, wearing adult diapers, and multiple toilet trips can be stigmatizing. Negative stereotypes deriving from social and cultural ideas of bodily self-control, competence, social interruptions, urination as a private act, masculinity and femininity, and sexuality [12] becomes associated, resulting in stigmatizing effects that devalue and discredit these older men [36]. Stigmatized individuals also internalize the strict and harsh social ideals as a code for their own self-evaluation and eventually result in the devaluation of self, which is also known as self-stigma [20,37]. People who internalize stigma related to urinary problems may be less likely to seek professional help for such problems to avoid disclosing their identity and being judged [12,20,35,38]. The impact of self-stigma has been extensively documented in research on HIV+, mental health, and physical disabilities, but little has been studied with regards to urinary problems in spite of the relevance to older men. Help-seeking behavior has been an issue of concern for helping professionals because help-seeking rates for older men with urinary problems are prevalently low [17,39,40,41,42].

Individuals distressed by urinary problems may be more likely to have negative self-perceptions, particularly in sexual relationships. Previous studies found negative influences of urinary problems on the self-esteem of patients [17,20], sleep quality [27], and the sexual and marital satisfaction of their spouses [21]. No study has compared the levels of self-esteem in sexual relationships between individuals distressed and non-distressed by urinary problems.

The present study investigated the proportion of urinary problems and distress due to urinary problems in the last six months and compared the levels of a range of psychosocial variables between the groups with and without distress due to urinary problems in a sample of Chinese male retirees in Hong Kong. This study aimed to bring to the surface the potential adverse impact of urinary problems on a range of psychosocial status, including mental health, coping self-efficacy, fatigue, and self-esteem in sexual relationships, so that more knowledge on the topic could be made available to helping professions and there is an opportunity for the participants to talk about this tabooed topic that might have disturbed them for some time. It is hypothesized that the group distressed due to urinary problems would have higher levels of fatigue symptoms and self-stigma of help-seeking and lower levels of mental health, self-esteem in sexual relationships, and coping efficacy than the group without distress due to urinary problems.

## 2. Methods

### 2.1. Participants

Members of a district-wide pilot project on retired men were invited to take part in this study. Inclusion criteria included a) men aged 65 or above; and b) self-claimed to be retired. Exclusion criteria included a) persons with a known diagnosis of dementia; and b) persons with a known medical condition in relation to the urinary system (e.g., prostate cancer or kidney disease). From the membership list of 200, 139 members (69.5%) were successfully interviewed face-to-face by trained male interviewers with their informed consent. Ethical approval of the study was obtained from the Baptist University of Hong Kong in 2011.

### 2.2. Survey Instruments

A structured questionnaire was used in the study, which contained socio-demographic characteristics, the participants’ experience with urological and other health-related items, and five validated psychological instruments to measure mental health, self-stigma of seeking help, fatigue symptoms, coping self-efficacy, and self-esteem and relationships. The details of the health-related items and the psychological scales are as follows:

The single-item questions were used to assess the presence of urinary problems, urinary incontinence in the past six months, coping with urinary incontinence/nocturia, sleep problems due to nocturia, distress due to urinary problems, health satisfaction, and life satisfaction, respectively. These single items were created and used after a focused-group discussion with some participants. These items are considered relevant and indicative of one’s personal situation in relation to urinary problems. In a practical sense, the use of these items shortens the length of the interview time, making the interview more acceptable to the participants.

*General Health Questionnaire-12 (GHQ-12)* [43,44]: This is a screening measure used for identifying people suffering from mental health problems within a general population. It contains 12 questions, with a total score ranging from 0 to 36. Higher total scores indicate poor mental health. The Cronbach alpha ranged from 0.73 to 0.96.

*Self-stigma of Seeking Help (SSOSH)* [20]: This is a 10-item scale for assessing a respondent’s self-stigma, which is an important factor in people’s decisions not to seek professional help. Items are rated on a Likert scale, ranging from 1 (strongly disagree) to 5 (strongly agree). Higher scores indicate higher levels of self-stigma of seeking help. The Cronbach alpha ranged from 0.86 to 0.90. 

*Fatigue Symptom (MFSI-SF)* [45]: This multidimensional scale contains 30 items for measuring 5 fatigue subscales: general, physical, emotional, and mental fatigue, and vigor. Items are rated on a Likert scale, ranging from 0 (never) to 4 (very often). A total score can also be calculated for representing the overall fatigue condition. Cronbach alpha ranged from 0.79 to 0.86.

*Coping Self-efficacy (CSE)* [46]: This is a 13-item scale for measuring a person’s perceived ability to cope effectively with life challenges. It contains three factors: the use of problem-focused coping, the ability to make unpleasant thoughts go away, and to gain support from friends and family. Anchor points on the scale were 0 (cannot do at all), 5 (moderately certain can do), and 10 (certain can do). The Cronbach alpha ranged from 0.80 to 0.91.

*Self-esteem and Relationship (SEAR)* [47]: This scale contains 14 items for measuring a respondent’s sexual relationship and confidence. The items resolve into two domains: Sexual Relationship (eight items) and Confidence (six items), the latter comprising the Self-Esteem (four items) and Overall Relationship (two items) subscales. Response options include almost always/always, most times (much more than half the time), sometimes (about half the time), a few times (much less than half the time), and almost never/never. The Cronbach alpha ranged from 0.75 to 0.90.

### 2.3. Data Analysis

Descriptive statistics were used for compiling frequency tables for socio-demographic characteristics, as well as items related to urological problems experienced by the participants. Differences in the socio-demographic characteristics and scores obtained from the psychological scales between participants with and without distress due to urinary problems in the past six months were tested using independent t-test. The level of significance was set at *p* < 0.05. Statistical analysis was carried out using the statistical package of SPSS 16.0.

## 3. Results

### 3.1. Participant Characteristics

Among 139 participants, the majority were aged below 65 (62.6%), with a mean age of 63.5 (SD = 4.9). About 40% were within the normal range of BMI; 3.6% were overweight and 31.6% were considered (morbidly) obese. The majority were married or cohabited (84.9%), living with family or friends (87.8%) and were not in gainful employment (82.0%). Only 8.6% and about one-third (34.5%) of them were current smokers and drinkers, respectively. More than one-third of them (36.0%) were sexually inactive and allegedly had not had sex with anyone in the past twelve months (Table 1).

The group who felt distressed due to urinary problems had a higher proportion of normal range BMI (*p* = 0.01), more were not in gainful employment (*p* = 0.01), and more participants were sexually inactive in the past twelve months (*p* = 0.01) than the non-distressed group.

∞ *p*-value for either independent group T-test or Chi Square test of association; * *p* < 0.05.

### 3.2. Urinary Problems of the Participants

As shown in Table 2, one-third (34.5%) of the participants reported that they suffered from urinary incontinence due to not being able to get to the toilet in time. Over half (59.6%) reported that they could not sleep well due to nocturia. Moreover, 41.1% reported that they experienced little or severe distress due to the above urinary symptoms.

### 3.3. Associations between Distress Due to Urinary Problems and Psychological Status

When comparing respondents who were and were not in distress due to urinary problems, most of the scales show significant differences, except for the self-stigma of seeking help (Table 3). Particularly, respondents with distress due to urinary problems had poorer mental health (*p* < 0.001). They suffered more severe fatigue symptoms in all aspects measured by the fatigue symptom scale, including the general (*p* < 0.001), physical (*p* < 0.001), emotional (*p* < 0.001), mental (*p* < 0.001), and vigor scales (*p* = 0.01) and the total score (*p* < 0.001). As indicated by the statistics of the coping self-efficacy scale, the abilities to make unpleasant thoughts go away (*p* = 0.05) and to gain support from friends and family (*p* = 0.03) were also significantly weaker for those who felt distressed than those who did not feel distressed due to urinary problems.

For those who had any sexual relationships in the past twelve months (n = 89), those who felt distressed due to urinary symptoms experienced lower satisfaction in all dimensions of self-esteem and sexual relation. Significant differences between the two groups were found in the dimensions of sexual relation (*p* = 0.02) and overall total score (*p* = 0.04).

## 4. Discussion

Although the participants under study were generally healthy, mostly non-smokers, non-drinkers, married, and living with family members, one-third of the participants reported urinary incontinence and nocturia and 40% of all participants found the problem(s) to be disturbing. This suggests urinary symptoms, especially in their mild forms, might have been more common than was noticed [4,48,49,50,51,52]. Similar to other studies, near to half of the participants (46%) did not seek [53] or delayed seeking medical advice [42,54]. The gradual onset nature of the urinary problems might have accounted for some of these cases but is less able to explain the failure to seek professional help even when the men felt distressed by the symptoms. This might have to do more with the attribution to and acceptance of ageing, taking these symptoms as part of the “natural” process [17]. The expected embarrassment and the possible threat to masculinity caused by disclosing one’s urinary symptoms and their associated problems, especially erectile dysfunction (ED) problems, might also account for their silence. Previous research has highlighted the importance of self-management strategies and interventions to improve the quality of life of people with urinary problems [26,55]. However, from a psychological perspective, related education workshops and campaigns to reduce the potential maladaptive cognitive beliefs about urinary problems and their symptoms are needed in order to promote help-seeking behaviours and early treatment for urinary problems [17]. Currently, in the field of urology, more psychosocial research is warranted [10]. Research has indicated that the relationship of depression or anxiety with urinary problems may be bidirectional [10,27]; the specific emotions associated with urinary problems such as low self-esteem, embarrassment, and self-blame may also offer implications towards mental health [31]. Research delineating the mechanism would further inform whether psycho-education is sufficient or whether tailored psychological intervention would be more beneficial [10].

The distressed group reported significantly poorer psychological health status, including poorer mental health, more fatigue symptoms, and poor coping self-efficacy. These results are consistent with previous studies on the association between urinary problems and mental/emotional health problems [5,11,19,23,24,25]. However, they did not differ much from the non-distressed group in seeking help. This may indicate that the distressed participants were coping with the problems at a cost to themselves, both mentally and physically. It echoed the findings that male participants interpreted urinary problems as deficiencies, rather than symptoms, and were therefore less likely to report these problems to general practitioners [54]. A general check-up may serve as a good starting point from which more sensitive issues could be further discussed. Health education pertinent to specific groups and age groups should be promoted, and knowledge of male psychology should be shared with the helping professions so that they may become effective referral agents. Arrangement of services to retirees should also address the taboo and sensitivity of the urinary issues, apart from financial and accommodation concerns. Male volunteers of a similar age might be a resource at the waiting lobby for persons who come because of urinary troubles. These volunteers may even be invited to share how they feel and experience in the related course of medical education and contribute to the enhancement of professional competence. There are many different possibilities of interventions to supplement public health issues and in this case, urinary distress among retired men at the community level. As always, public health issues in the community are not just concerns inside the consultation room and have to be handled in a comprehensive manner. It is worth noting that the groups “little distress” and “severe distress” were combined in the analyses and most cases had little distress due to urinary problems. Thus, it is plausible that those who experienced little distress did not perceive the need to seek help for urinary problems.

Although there was no significant difference in self-esteem and confidence between the groups of sufferers and non-sufferers of urological symptoms, men who felt distressed with urinary symptoms were also significantly less satisfied with their own sexual relations. This is the first study that explored the impact of urinary problems on self-perceptions in sexual relationships. This new piece of information is valuable for general practitioners and urological specialists. Probing sensitively and sensibly into a patient’s sexual satisfaction and self-esteem in sexual relationships may be necessary, even when there may not be an initial self-report of erectile dysfunction. Feeling distressed with urinary symptoms warrants respectful handling and when the patient is ready to talk about the related troubles of his sexual life (e.g., sexual relation and sexual satisfaction), the impact should also be explored. There are certainly psychosocial aspects of the medical condition to be addressed.

While it is clear that a physical health condition like urinary symptoms would have a negative impact on the psychological and social health of oneself, it is less clear about the exact interaction between physical and social factors or the pathway leading to the decision of seeking/not seeking medical help. Future studies are needed to differentiate who are more influenced by what in that decision making process.

## 5. Limitations

There is a number of limitations with sample size, sampling and recruitment, measurement vigor, and statistics. The sample size is small and the sampling design is not a random one. It makes the findings suggestive rather than conclusive and caution has to be taken in interpreting the results, though the sensitive data on urinary and sexual activities are always difficult to collect in view of strong taboo in Asian culture to talk about personal issues that may imply a threat to their sense of masculinity. Since the level of distress is highly subjective and difficult to compare within persons and between persons, categories with less contention (i.e., no, little, severe) were used instead of more varied choices of “Some/moderate/considerable”. This may have a higher chance of interpretational biases. The current response set of “no distress/little distress/severe distress” is on one hand easier for participants, however it also runs the risk of losing important information on the intensity of the problem. The urinary symptoms and fatigue symptoms experienced in some participants, in some cases, can be a symptom of an (possibly undiagnosed) underlying condition, which requires more research and attention from health care providers. In addition, the face-to-face interview of this study may have induced issues with social desirability and embarrassment and thus, participants may have made their responses less extreme. The subjects are those who admitted to being retirees and naturally exclude many who are at retirement age, yet who are still working full-time. The subjects’ physical and psychological health profile may be very different from that of the non-sample subjects. The non-random sampling and the recruitment through a retiree social club may have included those who are healthier and more active. The exclusion of those with medical conditions related to the urinary system, like prostate cancer case and dialysis cases, may also reinforce biased sampling. Therefore, caution has to be taken in inferring to a wider population. The one-off data collection also inevitably provides limited information on the actual development of urinary symptoms and prevents the charting of the changes over time. A longer interval (e.g., 5 years or 10 years) follow-up may be better able to capture the changes over time and may provide a fuller picture of symptom development, help-seeking and coping, and associated male psychology. Finally, a qualitative interview would be helpful in the future to supplement contextual information about the sensitive issues if subjects are willing to share.

## 6. Conclusions

Judging from the relatively younger age and generally healthy profile of the participants, this study portrayed the psychosocial issues of those who have mild to moderate urinary symptoms. It is this aspect of the study that contributes to our understanding of the early stage of urinary problems in retired men. Urinary symptoms in their milder form should not be overlooked or handled without a psychosocial perspective. How to translate this understanding into better sensitivity and responsive health and social care remains a challenge for helping professions and service providers.

## Figures and Tables

**Table 1:** Socio-demographic Characteristics.

		Not Distressed (N = 82)	Distressed (N = 57)	Total (N = 139)	*p*-Value ∞ (independent group *t*-test)
Member Type	Very active	5 (6.1)	1 (1.8)	6 (4.3)	0.14
	Active	50 (61.0)	29 (50.9)	79 (56.8)	
	Non-active	27 (32.9)	27 (47.4)	54 (38.8)	
Age	Mean ± SD	62.9±4.8	64.5±4.9	63.5±4.9	0.06
	≤64	55 (67.1)	32 (56.1)	87 (62.6)	0.42
	65-74	26 (31.7)	24 (42.1)	50 (36.0)	
	75-84	1 (1.2)	1 (1.8)	2 (1.4)	
BMI	Mean ± SD	24.3±3.0	23.0±3.7	23.8±3.4	0.02*
	Underweight < 18.5	2 (2.4)	3 (5.3)	5 (3.6)	0.01*
	Normal 18.5–22.9	23 (28.0)	32 (56.1)	55 (39.6)	
	Overweight 23–24.9	26 (31.7)	9 (15.8)	35 (25.2)	
	Obese 25–29.9	27 (32.9)	11 (19.3)	38 (27.3)	
	Morbidly Obese ≥ 30	4 (4.9)	2 (3.5)	6 (4.3)	
Marital Status	Married/Cohabited	68 (82.9)	50 (87.7)	118 (84.9)	0.53
	Separated/Divorced	5 (6.1)	4 (7.0)	9 (6.5)	
	Widow	6 (7.3)	1 (1.8)	7 (5.0)	
	Single	3 (3.7)	2 (3.5)	5 (3.6)	
Employment status	Full-time employed	5 (6.1)	0 (0.0)	5 (3.6)	0.01*
	Part-time employed	16 (19.5)	4 (7.0)	20 (14.4)	
	Retired	61 (74.4)	53 (93.0)	114 (82.0)	
Living with others	Living alone	10 (12.2)	6 (10.5)	16 (11.5)	0.47
	Living in old age home	0 (0.0)	1 (1.8)	1 (0.7)	
	Living with family or friends	72 (87.8)	50 (87.7)	122 (87.8)	
Smoking habit	Never	47 (57.3)	36 (63.2)	83 (59.7)	0.75
	Quitted	27 (32.9)	17 (29.8)	44 (31.7)	
	Smoker	8 (9.8)	4 (7.0)	12 (8.6)	
Drinking habit	Never	32 (39.0)	16 (28.1)	48 (34.5)	0.22
	Quitted	21 (25.6)	22 (38.6)	43 (30.9)	
	Drinker	29 (35.4)	19 (33.3)	48 (34.5)	
Sex in the past 12 months?	Yes	60 (73.2)	29 (50.9)	89 (64.0)	0.01*
	No	22 (26.8)	28 (49.1)	50 (36.0)	

**Table 2:** Life satisfaction and urinary problems faced by the respondents (n = 139).

		Frequency	%
Are you satisfied with your living status? (Missing = 1)	Very satisfied	6	4.3
	Satisfied	111	80.4
	Unsatisfied	19	13.8
	Very unsatisfied	2	1.4
Are you satisfied with your health status?	Very satisfied	10	7.2
	Satisfied	88	63.3
	Unsatisfied	37	26.6
	Very unsatisfied	4	2.9
Urinary problem	No	92	66.2
	Self-suspected	27	19.4
	Diagnosed	20	14.4
Over the past six months, did you have urinary incontinence?	Never	91	65.5
	One to two times	24	17.3
	Sometimes	22	15.8
	Frequently	2	1.4
When you had urinary incontinence, have you taken any action to solve the problem? ^#^ (N = 48)	Seek help from medical professionals	26	54.2
	Self-medication	0	0.0
	No action	23	47.9
Over the past six months, you could not sleep well because of nocturia.	Never	56	40.3
	One to two times	28	20.1
	Sometimes	37	26.6
	Frequently	18	12.9
When you had the problem of nocturia mentioned above, have you taken any action to solve the problem? ^#^ (N = 83)	Seek help from medical professionals	37	44.6
	Self-medication	2	2.4
	No action	47	56.6
Over the past six months, did you feel distressed due to the above urinary problems?	No	82	59.0
	Little	49	35.3
	Severe	8	5.8

# More than one answer was allowed.

**Table 3:** Relationships between distress due to urinary problems and psychosocial status.

		Over the Past Six Months, Did You Feel Distressed Due to the Above Urinary Problem?				
		Little/Severe (n = 57)		No (n = 82)		*p*-Value (Independent Group *t*-test)
		Mean	SD	Mean	SD	
Psychological Health Status (score range: 0–36)	Total Score	4.25	3.06	2.09	2.11	<0.001**
Self-Stigma of Seeking Help (score range: 10–50)	Total Score	26.02	3.44	25.20	4.16	0.221
Fatigue Symptom (score range: 0–120)	General scale	8.18	4.91	4.15	3.89	<0.001**
	Physical scale	6.70	4.20	3.44	3.31	<0.001**
	Emotional scale	7.96	5.12	4.05	4.02	<0.001**
	Mental scale	8.42	3.92	5.22	3.82	<0.001**
	Vigor scale	11.82	4.75	14.24	5.35	0.007**
	Total score	19.44	19.09	2.61	15.66	<0.001**
Coping Self-efficacy (score range: 0–130)	Use problem-focused coping	37.18	9.71	39.44	11.58	0.229
	Stop unpleasant thoughts go away	23.23	8.06	26.07	8.40	0.048*
	Get support from friends and family	18.53	6.45	20.77	5.82	0.034*
Self-esteem and Relationship (score range: 14–70)		Little/Severe (n = 29)		No (n = 60)		
Domain 1: Sexual Relationship		45.26	19.30	54.46	15.53	0.018*
Domain 2: Confidence		54.25	19.21	60.83	15.70	0.089
Subscale 1: Self-Esteem		53.79	19.44	61.33	15.83	0.054
Subscale 2: Overall Relationship		55.17	21.98	59.83	18.09	0.327
Total score		49.11	18.46	57.19	14.31	0.044*

* *p* < 0.05; ** *p* < 0.01.
